# HUNCHEST projects—advancing low-dose CT lung cancer screening in Hungary

**DOI:** 10.3389/pore.2024.1611635

**Published:** 2024-05-09

**Authors:** Anna Kerpel-Fronius, Krisztina Bogos

**Affiliations:** ^1^ Department of Radiology, National Korányi Institute for Pulmonology, Budapest, Hungary; ^2^ Director of the National Korányi Institute for Pulmonology, Budapest, Hungary

**Keywords:** lung cancer, screening, LDCT, pulmonary nodules, implementation

## Abstract

Lung cancer, the leading cause of malignancy-related deaths worldwide, demands proactive measures to mitigate its impact. Low-dose computer tomography (LDCT) has emerged as a promising tool for secondary prevention through lung cancer screening (LCS). The HUNCHEST study, inspired by the success of international trials, including the National Lung Cancer Screening Trial and the Dutch NELSON study, embarked on the first LDCT-based LCS program in Hungary. The initiative assessed the screening efficiency, incorporating lung function tests and exploring the interplay between lung cancer and chronic obstructive pulmonary disease (COPD). Building upon this foundation, an implementation trial involving 18 Hungarian centers supported by the Ministry of Human Capacities demonstrated the feasibility of LCS within a multicentric framework. These centers, equipped with radiology capabilities, collaborated with multidisciplinary oncology teams, ensuring optimal patient pathways. However, a critical challenge remained the patient recruitment. To address this, the HUNCHEST 3 project, initiated in 2023, seeks to engage general practitioners (GPs) to reach out to eligible patients within a municipality collective of 60 thousand inhabitants. The project’s ultimate success is contingent upon the willingness of eligible individuals to undergo LDCT scans. In conclusion, the HUNCHEST program represents a crucial step in advancing lung cancer screening in Hungary. With a focus on efficiency, multidisciplinary collaboration, and innovative patient recruitment strategies, it endeavors to contribute to the reduction of lung cancer mortality and serve as a blueprint for potential nationwide LCS programs.

## Introduction

The year 1912 marked the beginning of formal documentation of lung cancer cases, with Isaac Adler publishing a review that identified 374 documented instances [1]. Fast forward to the present, and lung cancer annually claims the lives of 1.7 million people globally, with Hungary alone witnessing about 10,000 new cases each year [2].

Regrettably, by the time lung cancer becomes symptomatic, it often presents in an advanced or metastatic stage. Presently, surgery remains the sole curative option, but without early detection, only 15%–25% of cases are operable [3].

The disease has witnessed significant progress in the realm of early detection and prevention. This article focuses on the role of low-dose computer tomography (LDCT) in identifying the disease in its earliest, most treatable stages. While primary prevention through smoking cessation programs is essential to reduce new cases, secondary prevention, in the form of screening, plays a vital role in reducing mortality rates.

Early screening efforts initially centred on conventional radiography, utilizing chest X-rays (CXR) since they were widely accessible. In the 1960 s, several controlled trials were conducted, such as the Czechoslovakian and the Mayo Lung Project, which used chest X-rays, and the Johns Hopkins trial, which employed sputum cytology [4–6]. The final significant trial employing chest X-rays was the Prostate, Lung, Colorectal, and Ovarian trial, which followed over 150,000 patients for 13 years, but failed to show a reduction in mortality [7]. In Hungary, lung CXRs were a part of the fight against tuberculosis—the mandatory nature was later revoked, but still a large proportion of adults view CXRs as part of a health check [8]. Nearly 1,000 lung cancer cases are still detected this way in a population of 10 million. These patients have approximately twice the number of resectable LCS than their symptomatically detected counterparts [9].

The true breakthrough came with advancements in medical imaging technology. In 1992, the Early Lung Cancer Action Project (ELCAP) was launched in the United States of Amerika, by Claudia Henschke and her team, with a focus on LDCT screening. Over 31,000 asymptomatic individuals were screened, resulting in the diagnosis of 484 lung cancers, 85% of which were at Stage I. This was the first large-scale trial to demonstrate the potential of LDCT screening in lung cancer [10].

In the United States, the National Lung Cancer Screening Trial (NLST), initiated in 2002, proved to be a game-changer. It was a control-armed, prospective trial, involving 53,454 high-risk individuals. The results announced in 2013 revealed a 20% mortality reduction in the LDCT screening arm [11]. This compelling data led to the United States Preventive Services Task Force (USPSTF) in 2013 recommending LDCT lung cancer screening for individuals between 55–80 years of age with a smoking history of at least 30 pack-years who are active smokers or quit within the last 15 years—based on clinical data. This was modified in 2021 to include individuals as young as 50 years of age with a 20 pack-year history [12]. Medicare coverage was provided for at-risk individuals, although uptake remained low [13].

In Europe, the Dutch-Belgian Randomized Lung Cancer Screening Trial (NELSON) is the largest concluded lung cancer screening study to date. Data presented in 2018 showed a 26% reduction in mortality for high-risk males, and even more significant benefits for women. The introduction of an “indeterminate for cancer” category reduced the number of false positives in comparison to the NLST [14].

In 2015, the European Respiratory Society and the European Society of Radiologists published a joint statement followed by the European position statement on lung cancer screening in 2017 [15, 16]. These documents emphasize the importance of risk stratification, patient education, quality assurance, and a clear pathway for managing screen-detected nodules.

In Hungary, the first prospective LDCT lung cancer screening project started as early as in 2013. In this article the authors present a brief review of the results of the finished screening projects, and introduce the ongoing LDCT-LCS projects.

## HUNCHEST I

The HUNCHEST (Hungarian Chest Screening) pilot initiative, spearheaded by the National Korányi Institute for Pulmonology in Budapest, sought to evaluate the efficacy of LDCT in detecting lung cancer in asymptomatic individuals, regardless of established risk factors [17].

Initially conceived as a single-center study, the program aimed to establish screening protocols, reporting mechanisms, and ensure comprehensive patient follow-up. In 2015, additional thoracic centers specializing in lung cancer imaging joined the initiative, bringing the total to six active centers contributing to screening efforts. Each center employed adaptable recruitment strategies, leveraging media campaigns, websites, posters, newspaper advertisements, and informational leaflets to encourage voluntary participation.

The study encompassed individuals undergoing the first screening round between October 2013 and January 2020. Inclusion criteria targeted asymptomatic individuals aged between 50 and 79 years of age, irrespective of known risk factors. Participants with a history of smoking received smoking cessation counseling at recruitment. Exclusion criteria, in line with the NELSON trial and study protocol, excluded individuals with specific health conditions, self-reported moderate or poor health, permanent oxygen therapy needs, body weight of 140 kg or more, a history of cancer within the past 5 years, previous lung surgery, or chest CT examinations within the last 2 years. Written informed consent was mandatory, and those unable to provide it were excluded. Participants were categorized based on smoking habits and comorbidities.

The HUNCHEST program included lung function tests (spirometry) for all applicants to identify undiagnosed chronic obstructive pulmonary disease (COPD), with specific criteria for diagnosis and severity assessment.

LDCT protocols were tailored to the scanner model, with scans conducted during suspended maximal inspiration in a single breath-hold, covering the entire lungs. Radiation exposure was controlled, and imaging conditions were standardized across sites. Two independent radiologists read all scans with semiautomatic segmentation of nodules and manual measurements conducted as needed. The Siemens SyngoVia MM Oncology Lung Computer-Aided Detection (CAD) software played a crucial role in matching previously detected nodules and calculating the volume doubling time (VDT) for nodule growth assessment.

Nodules were categorized based on their VDT into likely benign (VDT >600 days), suspicious (VDT <400 days), inflammatory (VDT <40 days), and indeterminate (VDT between 400 and 600 days), necessitating further evaluation.

The number of screen-detected malignancies and positive predictive values in the study aligned with internationally published studies. Similarly to the NELSON protocol, the study incorporated not only positive/negative categories, but also an indeterminate category, optimizing nodule management. A web-based structured reporting platform facilitated clear pathways post a positive screen, enabling cost-effectiveness calculations and providing vital data for endorsing a nationwide risk group-based screening program.

Notably, the trial included never-smokers in its cohort, both with and without COPD as a comorbidity. This pioneering aspect positioned the initiative among the first to comprehensively evaluate Caucasian never-smoker participants concerning COPD within the context of a low-dose CT screening project. Despite acknowledged limitations, including the absence of a detailed evaluation on why non-smokers were sensitized for screening, the trial’s outcomes offered a unique vantage point for assessing the cost-effectiveness within this specific subgroup. Participants who tested positive during screening were referred to specialized pulmonologists. These experts assessed the necessity for further diagnostic measures or treatments based on available guidelines. These measures included full-dose contrast-enhanced chest or comprehensive staging CT scans, PET-CT scans, bronchoscopy, transthoracic needle biopsy (TNB), or video-assisted thoracoscopic surgery (VATS). The study meticulously documented the diagnosis, stage, pathology, and treatment plan for each case of lung cancer.

In conclusion, 1.5% of participants were diagnosed with histologically proven lung cancer, a percentage consistent with international data within the study population, ranging between 0.8%–2.2%.

The HUNCHEST study provided answers to health economy questions, revealing the annual costs of both screened and unscreened populations [18]. In the initial year, lung cancer screening with LDCT incurs an additional annual cost of approximately 3.3 billion HUF. By the 5th year, there is a yearly surplus cost of 1.9 billion HUF, considering a 10% participation rate of the affected population. The direct additional costs associated with screening amount to roughly 2.6 billion HUF per year. In the first 3 years of screening, the therapy for newly detected patients is more expensive than for those without screening. However, in the 4th and 5th years, the cost of treating later-stage, more expensive, and less effectively managed patients in the unscreened group surpasses the therapeutic cost of screened patients. By year ten screening is not only cost effective, but cost-saving.

## HUNCHEST-II

The HUNCHEST II extended implementation study model examination was launched in 2019 with the proposal and support of the State Secretariat for Healthcare of the Ministry of Human Resources. The study included 18 centers, following a uniform protocol, applying the same patient follow-up scheme after positive screening results. The goal was to shed light on how a LDCT lung cancer screening program could be expanded nationwide. The key question during the study was whether it could be proven that lung cancer is more likely to be detected in symptom-free, early stages among 50–74-year-olds who are current or former heavy smokers participating in the program. Another aim was to conduct a cost-effectiveness and budgetary impact analysis based on the real-life data obtained during patient care in HUNCHEST II [19].

A cornerstone of the study was the uniform nodule tracking protocol, with the expectation of minimizing regional healthcare disparities. The recommendation and implementation of smoking cessation support for active smokers was carried out according to the specified professional guidelines using the methods outlined for smoking cessation support. The task of expediting the examination of highlighted patients fell under the responsibility of the territorial pulmonary department. Special diagnostic teams dedicated to handling the diagnostic pathway of nodules detected during lung cancer screening had to be established at the examination centers. Initially, these teams were closely associated with the oncology multi-disciplinary team (MDT) and in cases of confirmed lung cancer diagnosis, the routine MDT consultation decided on the patient’s further course. (Internationally, it is recommended to establish a MDT for discussing solitary pulmonary nodules—approximately 70% of the cases identified are ultimately non-tumorous, and unnecessary invasive investigations can be reduced through MDT discussions).

During the examination, the designated radiologist at each center evaluated LDCT images on-site or through remote reporting. In addition, a central core Computer-Aided Detection (CAD) system provided by Aidence, the Veye Lung software provides the necessary secondary reporting. The images are automatically sent from the examination center’s PACS system to CoreCAD for central processing, and almost in real-time (expectedly within 5–10 min), CoreCAD provides a diagnosis established by the computer. The radiologist had this data available by the time she started the reporting process -this also replaced the need for the resource-intensive dual radiologist reporting.

AI tools have become a necessity in LCS programs—the correct volume measurements require computer assistance, and the correct assessment of VDT also relies on CAD system. These cannot however be applied as first readers, as it suggests in the name these “aid the diagnosis.” Deep learning (DL) systems are also developed in the field of LCS—in a recent metaanalysis, their specificity was 0.63 and sensitivity 0.93. The biggest question behind these systems that it is unclear how the machine calculates these results, so while promising, they are not yet accepted as part of non-study based screening projects [20].

Each center reported screenings to the National Korányi Institute for Pulmonology through an online data submission platform designed for this purpose, in compliance with GDPR regulations. The online interface is based on the tuberculosis surveillance system recorded by the National Korányi Institute for Pulmonology (OKPI) Methodology Department but is separate from it. Not only did it monitor the completion and results of controls in indeterminate screenings, but also, in the case of a positive screen it recorded the results of all necessary investigations. In the event of a lung cancer diagnosis, the histological type, stage, and the therapy suggested by the MDT was also documented. In the event of an alternative diagnosis, diagnosis and a brief description of the diagnostic pathway leading to it (bronchoscopy, PET/CT, biopsy, surgical intervention) was also noted.

In the clinical trial, data from more than 4,000 individuals were analyzed, with an average age of around 61 at the time of enrollment. Among the participants, the baseline LDCT examination result was negative in nearly 75% of all cases, and positive in 4%. The remaining group required LDCT follow-up, predominantly resulting in negative findings. In cases with positive results, every individual underwent a pulmonary specialist examination. Those with suspected tumors were appropriately referred to the local MDT for further assessment according to the protocol. Ultimately, 61 individuals were confirmed to have malignant lung tumors based on histopathology and/or clinical and radiological images.

Comparing the stage-wise distribution of new lung cancer patients participating in the HUNCHEST II program with those treated in the National Korányi Institute for Pulmonology (same time frame, same age range, confirmed smokers), it became evident that the HUNCHEST II study more frequently succeeded in detecting lung cancer in early stages. According to OKPI data, nearly 70% of patients presenting with symptoms were inoperable, while in HUNCHEST II, this was only the case for 20% of screen-detected tumor patients (Table 1).

**TABLE 1:** Comparison of histological subtypes of screen detected and incidental lung cancer.

	All^d^	Adenocc	Squamousc.cc	Small cell Cc	Other^e^
HC1^a^	29	18 (62.1%)	7 (24.1%)	2 (6.9%)	2 (6.9%)
HC2^b^	48	27 (56.2%)	15 (31.2%)	3 (6.2%)	5 (10.4%)
OKPI^c^ data 2022	NA	50%	23%	13%	5%
OKPI^c^ data 2019	NA	47%	24%	14%	6%

^a^ HUNCHEST I.

^b^ HUNCHEST II.

^c^ National Korányi Institute for Pulmonology.

^d^ In case of HC2 26 patients withdrew from follow-up, exact histological data cannot be collected, therefore excluded.

^e^ In both 2019 and 2022 9% of OKPI patients had no exact histological classification.

Comparing the statistics between HUNCHEST I and HUNCHEST II reveals several differences in participant characteristics during the 1st round of screening. The average age in HUNCHEST I was 63.2, slightly higher than the average age of 61.3 in HUNCHEST II. The female percentage among current smokers was similar in both studies. The number of former or never smokers was comparable between the two studies, with slight differences in age and gender distribution. The prevalence of COPD as a comorbidity was higher in HUNCHEST I (18.6%) compared to HUNCHEST II (13.2%) This was due to the proactive screening for COPD in the first study with standard lung function testing, whereas in HUNCHEST II self-reporting of the disease was noted. In summary, HUNCHEST II involved a larger and slightly younger cohort, with a lower prevalence of COPD, as a comorbidity. The gender distribution varied slightly, and HUNCHEST II had a higher number of participants with a positive screen in the 1st round compared to HUNCHEST I.

The examination of lung cancer histological subtypes within the screening programs HUNCHEST I (HC1) and HUNCHEST II (HC2), alongside data from OKPI, unveils intriguing variations. In the screening-focused HC1, adenocarcinomas prevailed at 62.1%, contrasting with HC2 at 56.2%. OKPI reported 50% in 2022 and 47% in 2019. HC2 exhibited a higher frequency of squamous cell carcinomas (31.2%) compared to HC1 (24.1%), closely mirroring OKPI’s 23% in 2022 and 24% in 2019—the lower incidence of this subtype in HUNCHEST I is possibly due to the fact that this program included never smokers, where squamous cell carcinomas are not common. For small cell carcinomas, HC1 was at 6.9%, HC2 at 6.2%, OKPI 2022 at 13%, and OKPI 2019 at 14%—the number of small cell carcinomas are usually lower in screening programs than in real life data, due to its more aggressive nature—the tumor grows much faster, thus making screening for it difficult. Other subtypes (including large cell tumors and carcinoids) constituted 6.9% in HC1, 10.4% in HC2, 5% in OKPI 2022, and 6% in OKPI 2019. These observations underscore the nuanced prevalence of lung cancer subtypes in screening programs, emphasizing the importance of considering diverse datasets in clinical and research contexts, particularly in the context of screening efforts (Table 2).

**TABLE 2:** Comparison of characteristics of participants in 1st round of screening in HUNCHEST I and II.

	All participants		People who currently smoke		Former or never smokers		Positive screen in 1st round	
	HC1^a^	HC2^b^	HC1	HC2	HC1	HC2	HC1	HC2
Number of participants	1890	4,215	870	3,284	1,020	931	70	174
Age	63.2	61.3	64.5	60.6	62.1	63.1		
Females	1,071 (56.7%)	2,254 (53.5%)	0,564 (55.3%)	1811 (55.1%)	507 (58.3%)	443 (47.6%)	38	97
COPD^c^ as comorbidity	351 (18.6%)	556 (13.2%)	258 (24.3%)	439 (13.4%)	103 (11.8%)	117 (12.6%)	19	34

^a^ HUNCHEST I.

^b^ HUNCHEST II.

^c^ Chronic obstructive pulmonary disease.

## Lung cancer screening projects in central Europe

In the past decade more and more European initiatives have started, most of them pilots—including Italy (MILD) and France (CASCADE), to name a few [21]. In the UK, regional programs have developed in such an extent, that today the Targeted Lung Health Checks are covering England by 2024 [22]. In 2020 Croatia was the first European country to roll out a nationwide screening project, with enrollment standing at over 29 thousand as the end of 2023 [23]. Historically Poland has a long standing history with LCS starting in 2008—today Poland has also started a nationwide project, based on the voivodeship system [24]. In the Czech Republic the nationwide system is based on pulmonologist, they refer patients in case of existing risk factors to the radiology departments [25]. These efforts reflect a comprehensive approach to lung cancer prevention and early detection across Europe. In Austria, Slovakia, Slovenia, Romania and Serbia no nationwide pilots were rolled out as of date, smaller studies such as the Vojvodina project in Serbia have been established, or in case of Slovakia, a comprehensive white paper has been formulated. Many of these countries, however, are part of the SOLACE project, thus implementation might start in these countries too [26–29].

## Ongoing programs

### HUNCHEST-III

In anticipation of a potential nationwide screening program, further studies are still necessary. The HUNCHEST I and II programs have provided compelling evidence supporting the cost-effectiveness of LDCT lung cancer screening within the appropriate risk group in Hungary. Notably, these initiatives were characterized by voluntary and opportunistic screening methodologies, focusing on modeling patient pathways post-screening rather than elucidating the routes leading to screening. Recognizing the significance of clarifying pre-screening patient journeys, we recommend a more nuanced approach by modeling primary care patient selection within a more confined population.

The primary dilemma facing lung cancer screening programs is their departure from age-specific screening, unlike other public health screenings, adopting a risk-based approach instead. Currently, there is available literature data regarding the effectiveness of screening individuals aged 50 (55)–75 (80) years with a significant smoking history (25–30 pack-years). Given this, the initiation of screening for this group is imperative. Unfortunately, obtaining precise smoking history is not readily available in most places, making the traditional invitation system based on residency records, as used in other screenings (e.g., breast cancer screening), unsuitable for lung cancer screening.

Illustrating the challenges faced in real-world, non-trial screenings, Kinsinger et al. conducted screening in Veterans Health Administration hospitals from 2013 to 2015, utilizing NLST criteria. Initially identifying 93,033 individuals meeting the initial screening criteria (age between 55–80 years, no serious comorbidities, and life expectancy of more than 6 months), nurses reviewed their histories, seeking individuals with at least a 30 pack-year smoking history, either current smokers or those who quit within the last 15 years. Due to missing data, 36,555 individuals were excluded, and an additional 38,395 lacked a smoking history suitable for screening. Among the remaining 18,083 individuals, doctors did not assess 13,084 cases, while 789 were deemed unsuitable for LDCT screening. Out of the remaining 4,246 patients, 1,794 declined screening, and eventually, 2,106 screenings were completed within the timeframe. Of these, 59.7% had a positive result based on NLST criteria, detecting suspicious lesions in 73 patients, ultimately confirming lung cancer in 31 cases. The false-positive rate was remarkably high at 97.5%. Additionally, 40.7% of patients had incidental findings, most commonly emphysema and coronary atherosclerosis [30].

Among those who participated in the screening, 1,120 individuals, according to NLST criteria, were expected to attend a follow-up examination after 1 year. Despite repeated invitations via written and telephone communication, only 870 individuals attended. This was less than 78%, significantly lower than the 95% recall success rate assumed in NLST’s public health calculations. These challenges underscore the complexities faced in implementing effective lung cancer screening programs, particularly in identifying and engaging the target population.

Thus, the aim of the final HUNCHEST project is to test prescreening pathways. To that effect a smaller, well described population based study was called for. Demographic overview encompassing the settlements affiliated with the “Budakörnyéki egészségprogram” Peri-Budapest Healthcare Program—Biatorbágy, Budajenő, Budakeszi, Herceghalom, Nagykovácsi, Páty, Perbál, Pilisjászfalu, Remeteszőlős, Telki, Tinnye, Tök collectively house approximately 60–65,000 residents. Within this demographic, an estimated 12,000 individuals fall within the 50–75 age bracket, with an anticipated 3,500–4,000 individuals exhibiting a substantial history of tobacco use. The overarching objective is to meticulously map the smoking history of all individuals within the specified age group, facilitating the identification and subsequent invitation of those at risk for LDCT screening.

The success of the screening program hinges upon the active involvement of general practitioners and their assistants. Their pivotal role involves assessing the smoking history of individuals aged 50–75 in their respective areas and discerning those deemed suitable for screening. Furthermore, at this juncture, a targeted smoking minimal intervention is administered. Following this initial phase, coordination with the Comprehensive Cancer Center’s coordinator ensues, whereby the collected information is meticulously recorded within the HUNCHEST platform.

We anticipate that 20% of screened patients will be recalled for a 3-month follow-up assessment based on our previous pilots. Based on the HUNCHEST program, the lung cancer identification rate is projected to range between 1.5%–2% in Hungary. This implies the potential detection of approximately 80 cases of lung cancer, with a substantial majority—around 70%—being identified in the early stages. This comprehensive approach holds the promise that approximately 65 patients may receive a genuine opportunity for long-term survival.

The HUNCHEST-III project is currently in the active recruitment phase, screening started in September 2023, preliminary results are excepted in early 2025.

### SOLACE

In 2022, the EU4Health project introduced a groundbreaking initiative aligned with Europe’s Beating Cancer Plan—the Strengthening the screening of Lung Cancer in Europe (SOLACE) project [31]. This innovative endeavor aims to streamline the implementation of lung cancer screening programs across Europe, ensuring equitable access for individuals from diverse social and economic backgrounds. Representing a significant stride in comprehensive lung cancer screening, SOLACE is dedicated to developing, testing, and disseminating tools that address identified obstacles and health inequalities in various European countries.

The primary goal of SOLACE is to provide a versatile toolbox for personalized approaches to lung cancer screening, applicable on both national and regional scales. The project specifically focuses on facilitating and supporting the structured implementation of LDCT lung cancer screening programs throughout Europe. By doing so, SOLACE aims to enhance the overall quality of lung cancer screening practices, while also improving accessibility, benefit-harm balance, and cost-effectiveness.

A key feature of SOLACE involves unprecedented collaboration among key stakeholders essential for designing, planning, and implementing sustainable lung cancer screening programs in member states. To ensure the lasting impact of the project, the proposal includes the establishment of the European Lung Cancer Screening Alliance (ELCSA).

Hungary actively participates in SOLACE with three centers, notably including the OKPI. Over the next 18 months, the project will test various recruitment strategies to measure their effectiveness in targeting different groups, with a special focus on the socio-economically deprived and on those with preexisting pulmonary comorbidities. Notably, the project places a particular emphasis on women, recognizing the insufficient data on lung cancer screening strategies in the female population.

## Conclusion

With the 3 HUNCHEST projects, we have modeled the three pillars of screening: the technical implementation of screening, the design of patient pathways post-screening, and the identification and invitation of high-risk patients, as seen in Table 3. The next step is to determine the feasibility of a potential public health screening. In the meantime, it may be advisable for the health administration to create a means allowing high-risk individuals to participate in LDCT screening once a year.

**TABLE 3:** Comparison of the different HUNCHEST studies.

	HUNCHEST I	HUNCHEST II	HUNCHEST III
aim	Demonstrating the feasibility of LCS in Hungary	Establishing patient pathways	Modelling population wide screening (estimated 4,000 at risk—determining efficacy of invitation methods)
date	2013-20	2019-22	2023-ongoing
criteria	50–79 both smokers and neversmokers	50–75 20 + pys	50–75 20 + pys
participants	1,890	4,215	TBA
Lung cancers found	1.5%	1.8%	TBA

Considering the future of large-scale LDCT screenings, key questions arise regarding the application of artificial intelligence (AI) and deep learning models to address human resource challenges. Furthermore, there is a need to determine additional biomarkers for individuals currently not in high-risk groups, such as non-smokers or young individuals, in order to develop screening in potentially identifiable risk groups, while adhering a thorough cost/benefit analysis.

The integration of AI and deep learning models in LDCT screenings presents a promising avenue for enhancing efficiency and accuracy in diagnosis. This technological advancement can alleviate human resource constraints by automating the analysis of LDCT results, and enabling quicker and more precise identification of potential tumors. Adequate training for healthcare professionals in collaboration with AI systems will be crucial to optimize this integration.

Identifying biomarkers beyond the current high-risk groups is essential. Research efforts should focus on exploring additional biomarkers that can aid in identifying low-risk groups more accurately. Factors such as genetic predispositions, environmental exposures, and other elements should be considered to refine the screening criteria and ensure a more targeted approach.

Conducting comprehensive cost/benefit analyses is imperative for shaping effective screening programs. Evaluating the costs against potential savings and improvements in patients’ quality of life will provide insights into the economic viability of such programs. Considering the long-term health and economic impacts is crucial in making informed decisions.

## Abbreviations

COPD, chronic obstructive pulmonary disorder; CXR, Chest X-ray; HC1, HUNCHEST I; HC2, HUNCHEST II; LCS, Lung cancer screening; LDCT, low dose computer tomography; MDT, multidisciplinary team; NELSON, Nederlands–Leuvens Longkanker Screenings Onderzoek (Dutch–Belgian lung-cancer screening trial); OKPI, Országos Korányi Pulmonológiai Intézet (National Korányi Institute for Pulmonolgy); NLST, National Lung Screening Trial; SOLACE, Strengthening the screening of Lung Cancer in Europe; VDT, Volume doubling time.
